# Comparison of AIMS2-SF, WOMAC, x-ray and a global physician assessment in order to approach quality of life in patients suffering from osteoarthritis

**DOI:** 10.1186/1471-2474-7-6

**Published:** 2006-01-26

**Authors:** Thomas Rosemann, Stefanie Joos, Thorsten Koerner, Joachim Szecsenyi, Gunter Laux

**Affiliations:** 1 Department of General Practice and Health Services Research, University of Heidelberg, Voβstr. 2, 69115 Heidelberg, Germany

## Abstract

**Background:**

Chronic diseases like osteoarthritis (OA) substantially affect different dimensions of quality of life (QoL). The aim of the study was to reveal possible factors which mainly influence general practitioners (GPs) assessment of patients' QoL.

**Methods:**

220 primary care patients with OA of the knee or the hip treated by their general practitioner for at least one year were included. All GPs were asked to assess patients' QoL based on the patients' history, actual examination and existing x-rays by means of a visual analog scale (VAS scale), resulting in values ranging from 0 to 10. Patients were asked to complete the McMaster Universities Osteoarthritis Index (WOMAC) and the Arthritis Impact Measurement Scale2 Short Form (AIMS2-SF) questionnaire.

**Results:**

Significant correlations were revealed between "GP assessment" and the AIMS2-SF scales "physical" (rho = 0.495) and "symptom" (rho = 0.598) as well as to the "pain" scale of the WOMAC (rho = 0.557). A multivariate ordinal regression analysis revealed only the AIMS2-SF "symptom" scale (coefficient beta = 0.2588; p = 0.0267) and the x-ray grading according to Kellgren and Lawrence as significant influence variables (beta = 0.6395; p = 0.0004).

**Conclusion:**

The results of the present study suggest that physicians' assessment of patients' QoL is mainly dominated by physical factors, namely pain and severity of x-ray findings. Our results suggest that socioeconomic and psychosocial factors, which are known to have substantial impact on QoL, are underestimated or missed. Moreover, the overestimation of x-ray findings, which are known to be less correlated to QoL, may cause over-treatment while important and promising targets to increase patients' QoL are missed.

## Background

About 10% of men and 18% of women 60 year and older suffer from symptomatic osteoarthritis (OA) [1]. Among people aged 75 or older it is the third most common reason to visit a primary care physician [2]. Due to an increasing life-expectancy as well as a constant increase in the Body-Mass-Index (BMI), which constitutes a principal risk factor for OA [3], the incidence of OA is expected to rise in upcoming years [4].

Usually, the General Practitioner (GP) is not only the first care provider but also sees the patient regularly during the course of disease [5]. Moreover, the GP is the physician who is most familiar with the social background of the patients. These socio-economic and psychosocial factors contribute substantially to the Quality of Life (QoL) of patients [6-8]. It is known that the treatment plans of GPs and specialists are quite similar, but also that there is a broad range of possible approaches to the disease by GPs [2,5,9]. However, previous findings indicate that GPs as well as specialists seem to have a perspective that is dominated by physical aspects of OA. Psychosocial aspects and their influence on QoL seem to be underestimated [10]. Moreover, it is known that radiographic changes and subjective complaints show very poor correlation, therefore it could be assumed that their contribution to physicians assessment of patients QoL may be limited. However, the extent to which psychosocial and radiological findings influence GPs assessment remains unclear. Interestingly, QoL is coming more and more into the focus of health care professionals and represents an increasingly important outcome parameter in many clinical trials [11]. Different questionnaires have been developed and validated to assess the impact of joint diseases on QoL. Assessing QoL in patients suffering from OA the most frequently used instruments are the McMaster Universities Osteoarthritis Index (WOMAC) [12,13], the Arthritis Impact Measurement Scale (AIMS) [14,15] and the Lequesne-Index [16].

WOMAC and Lequesne-Index both focus on physical effects of arthritis on mobility and physical activity and are limited to the lower limbs (hip and knee). The AIMS questionnaire originally developed by Meenan et al. in 1980 for rheumatoid arthritis is a more comprehensive tool, which includes in 78 items the five dimensions physical, affect, symptom, social interaction and role [14]. In 1997, Guillemin et al. developed a shorter version, the AIMS2-SF, containing 26 items, to reduce time effort and to increase acceptance among patients. In several validation studies, the AIMS2-SF, which is recently available in a German version [20], has proven to be a reliable and valid instrument to asses QoL of patients with rheumatoid arthritis and OA [17-19] and. Due to its comprehensiveness the AIMS2-SF gives insight into different dimensions of QoL in OA.

The aim of our study was to examine which dimensions of QoL of osteoarthritis patients are considered by GPs. To reveal factors influencing GPs' picture of patients QoL we estimated relationship to different assessment tools, AIMS2-SF, WOMAC, and x-ray.

## Methods

### Recruitment of patients

The study was approved by the ethical committee of the University of Heidelberg. From April to July 2004, 222 patients were approached in 21 primary care practices. Inclusion criteria for patients were: age over 18 years, meeting the criteria of OA according to the America College of Rheumatology (ACR) [21,22] and sufficient German language skills for understanding and answering the questionnaire. All patients had to be treated by the physician for at least one year. Additionally, the availability of a diagnose-specific radiograph, not older than six months at the date of questionnaire completion, was required. In all participating practices the patients were identified by ICD-10 codes in patient files and put on a list in alphabetical order. Patients from this list were contacted in consecutive order of appearance in the practice and informed about the option to participate in the survey. After giving their written informed consent they received the questionnaire and an envelope. The enveloped questionnaires were returned in a sealed box at the practices. Neither the GP nor the practice team had any possibility to see patients' answers.

Two of the addressed patients refused to complete the questionnaires due to time effort, so that 220 patients could finally be included. Enrolled patients received the validated German version of the WOMAC and AIMS2-SF questionnaire, each containing 5 point-Likert scales. In addition, patients received short written information about the aim of the study and were asked to personally complete the questionnaire without any assistance. For subgroup analysis data on school and professional education and present occupation were retrieved.

The patient's GP was asked to evaluate the severity of arthritis based on available radiographs, the patient's history and clinical examination based on classification criteria of the American College of Rheumatology. The GP's evaluation was scored on a visual analogue scale. The scale was organized in that manner that the results achieved ordinal values with 10 representing no limitation of QoL by arthritis to 0 with massive limitation of QoL. The x-rays were scored according to the criteria of Kellgren and Lawrence [13], Grade 0 = normal and Grade 4=massive alterations with close to complete reduction of the joint space. If only one joint was affected, the score for this joint was used. If different joints were affected, patients' highest radiological score was used.

### Statistical analysis

Data were analysed with the SPSS statistical package (version 12.0). In a standardisation process, the items of AIMS and WOMAC were recoded and transformed, so that results between 0 and 10 were yielded for all items with 10 representing the best health status and 0 representing the worst. This standardisation process was performed according to the recommendations of Guillemin (AIMS2-SF) and Bellamy (WOMAC), respectively. The radiological grading according to Kellgren and Lawrence was not adjusted. Descriptive analysis included mean and standard deviation separated for the mainly affected joint (hip or knee). In order to assess floor and ceiling effects the percentages of participants achieving the lowest and highest possible score were calculated.

Group comparisons were adjusted for sex and age and, in case of ANOVA, post hoc corrections according to Bonferoni were performed to avoid effects caused by multiple testing.

As a first estimation of factors influencing GPs' assessment of patients' QoL, we computed correlations of socio-demographic variables, AIMS2-SF, WOMAC, and Kellgren-score with the GP score. The correlations of the individual scales with the overall scores were computed via Spearman rang correlation. P-values are provided in order to show levels of statistical significance.

In order to identify factors independently associated with GP ratings of patient quality of life, we additionally performed a polytomous ordinal logistic regression analysis [23]. The purpose of this procedure is to model the dependence of an ordinal categorical response variable (here: GP ratings of patient quality of life) on a set of discrete and/or continuous variables (here: age, gender, education, physical, affect, symptoms, social interaction, function, stiffness, pain, Kellgren score).

### Subgroup analysis

Finally, subgroup analyses were performed by age, gender and level of education. A low level of education was defined by secondary school. More advanced degrees were considered high education. For the subgroup analysis the Student-t-Test for independent samples was used.

## Results

Table 1 reflects the baseline characteristics of the study sample, separated by gender. Main manifestation of OA in women was knee (60), while only 37 suffered mainly from OA of the hip. 78 men suffered mainly from OA of the hip, while in 45 cases men were mainly affected at the knee. This is in line with arthritis epidemiology showing that women suffer more from gonarthritis and men more from coxarthritis. Mean duration of disease among women was 11.3 years and 8.8 years among men. This difference was significant as well as the difference in age: men were older (49.8 vs. 43.7 years; p < 0.01).

**Figure 1:**
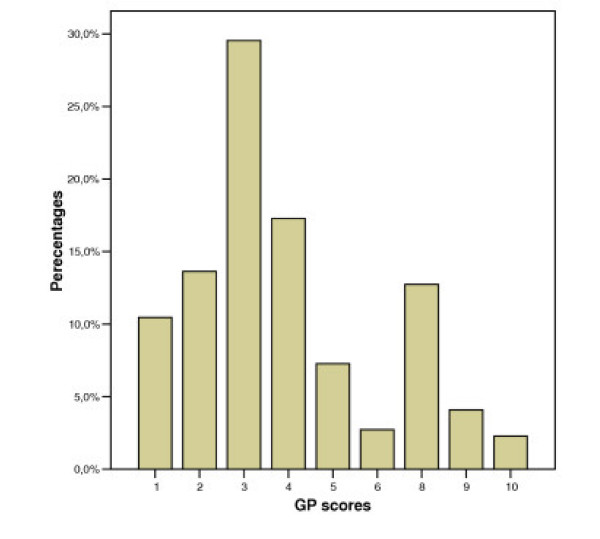


Table 2 shows the distribution of the Kellgren and Lawrence score according to the affected joint. ANOVA adjusted for age and gender with post hoc Bonferoni correction revealed no significant differences in Kellgren scores between patients suffering from osteoarthritis of hip or knee. Figure 1 shows the distribution of the GP scores in percentages.

In Table 3 the characteristics of the study sample are separated by the affected joint. Differences were assessed by adjusted ANOVA: Patients suffering from OA to the knee had a significantly higher BMI (27.9 vs. 26.3; p < 0.05; adjusted for sex and age), and suffered longer from the joint affection (11.2 vs. 9.3 years; p < 0.01; adjusted for sex and age). Regarding age, the hip and the knee group did not differ (ANOVA adjusted for sex).

Table 4 displays the descriptive statistics of the different assessments separated by the affected joint. As can be seen, the GP score as well as the Kellgren score did not differ significantly from each other. Regarding the AIMS2-SF, significant differences did occur in the scale "physical", in which a mean of 3.95 for the knee group indicated more limitation regarding physical aspects. The "symptom" scale indicated more impact due to pain in the knee group (p = 0.03). "Affect", "social" and "role" did not differ significantly. In accordance to this finding the WOMAC "pain" scale revealed significantly (p = 0.01) higher results for the knee group (5.93 vs. 5.21).

"Stiffness" also differed significant between knee and hip patients (5.48 vs. 4.96; p = 0.08).

**Figure 2:**
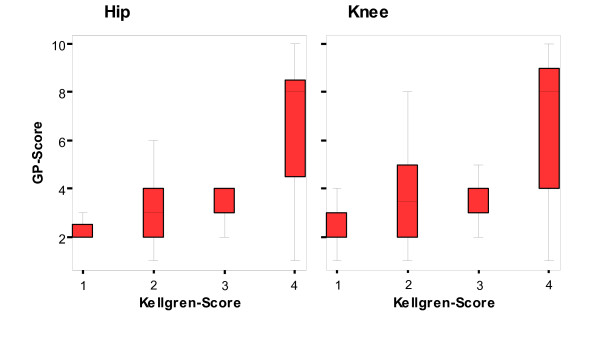


Figure 2 displays the GP score distribution in relation to the Kellgren score separated by hip and knee. As can be seen, the median of GP scores increases with the Kellgren score. Interestingly, in the hip and in the knee group the GP score achieved the widest range when patients were rated with a Kellgren score of two or four.

Table 5 displays statistically significant correlations for the GP assessment and the scale "physical" (rho = 0.495) and "symptom" (rho = 0.598) of the AIMS-questionnaire and good correlations (rho = 0.557) with the "pain" dimension of the WOMAC. The radiological grading according to Kellgren and Lawrence correlates quite well with the "symptom" dimension of the AIMS. All other correlations tended to be low, reaching the lowest values for the scales social and role of the AIMS2-SF.

Table 6 displays score distributions in demographic subgroups. Age was positively correlated with the impact of OA on QoL, reflected by higher scores in all instruments in the group aged over 60, despite the "affect" and "social" scale of the AIMS2-SF. Patients with lower education level achieved higher values in most scores. Women obtained higher values in most scores except for the "role" scale of AIMS2-SF. 111 patients were already retired from work, therefore numbers for the "role" scale were smaller.

Table 7 displays the results of the polytomous ordinal regression analysis that mirrors the dependence of the GP score on age, gender, education, on the AIMS2-SF scales "physical", "affect", "symptoms", "social" and on the WOMAC scales "function", "stiffness", "pain" as well as on the radiological grading according to Kellgren and Lawrence. Interestingly, in contrast to our bivariate comparisons, only "symptoms" and "Kellgren score" emerged as significant influence variables:

β^SYMPTOMS
 MathType@MTEF@5@5@+=feaafiart1ev1aaatCvAUfKttLearuWrP9MDH5MBPbIqV92AaeXatLxBI9gBaebbnrfifHhDYfgasaacH8akY=wiFfYdH8Gipec8Eeeu0xXdbba9frFj0=OqFfea0dXdd9vqai=hGuQ8kuc9pgc9s8qqaq=dirpe0xb9q8qiLsFr0=vr0=vr0dc8meaabaqaciaacaGaaeqabaqabeGadaaakeaaiiGacuWFYoGygaqcamaaBaaaleaaieGacqGFtbWucqGFzbqwcqGFnbqtcqGFqbaucqGFubavcqGFpbWtcqGFnbqtcqGFtbWuaeqaaaaa@37D3@ = *0.2588, 95% CI [0.03, 0.49], p = 0.0267*

β^KELLGREN
 MathType@MTEF@5@5@+=feaafiart1ev1aaatCvAUfKttLearuWrP9MDH5MBPbIqV92AaeXatLxBI9gBaebbnrfifHhDYfgasaacH8akY=wiFfYdH8Gipec8Eeeu0xXdbba9frFj0=OqFfea0dXdd9vqai=hGuQ8kuc9pgc9s8qqaq=dirpe0xb9q8qiLsFr0=vr0=vr0dc8meaabaqaciaacaGaaeqabaqabeGadaaakeaaiiGacuWFYoGygaqcamaaBaaaleaaieGacqGFlbWscqGFfbqrcqGFmbatcqGFmbatcqGFhbWrcqGFsbGucqGFfbqrcqGFobGtaeqaaaaa@3763@ = *0.6395, 95% CI [0.29, 0.99], p = 0.0004*

whereby β^j
 MathType@MTEF@5@5@+=feaafiart1ev1aaatCvAUfKttLearuWrP9MDH5MBPbIqV92AaeXatLxBI9gBaebbnrfifHhDYfgasaacH8akY=wiFfYdH8Gipec8Eeeu0xXdbba9frFj0=OqFfea0dXdd9vqai=hGuQ8kuc9pgc9s8qqaq=dirpe0xb9q8qiLsFr0=vr0=vr0dc8meaabaqaciaacaGaaeqabaqabeGadaaakeaaiiGacuWFYoGygaqcamaaBaaaleaaieGacqGFQbGAaeqaaaaa@2FF3@ represent the regression coefficient estimations according to the maximum likelihood method [24] for the underlying regression model. Obviously, the impact of "Kellgren score" appears to be stronger and is more significant in comparison to "symptoms".

## Conclusion

Physicians' assessment of patients' QoL is mainly influenced by two factors, pain and radiological findings. The results of the present study suggest that other factors, which are known to have an important influence on QoL of patients suffering from osteoarthritis such as socio-economic and psychosocial factors, are not sufficiently considered by the GPs.

### Strength and weaknesses of the study

To our knowledge, this is the first study exploring physicians' assessment of patients' QoL by estimating factors that may influence GPs. Some limitations have to be mentioned. In Germany more x-rays are taken in the care of patients with OA in comparison to other countries [25]. Therefore influence of x-ray findings on GPs may be higher than in other countries. Assessing the socio-economic status of patients by asking for the annual income is very uncommon in Germany, so that the educational level was used.

Without a doubt OA has an important impact on patients' QoL. This was revealed by multiple primary care based studies [26,27]. There is also strong evidence that QoL of patients suffering from chronic diseases is influenced by multiple individual factors as for instance support from family, the social situation, affect and mood [7,8,28-32]. Moreover, previous studies indicated that even physical disability can not only be explained by structural changes in the joint [6,29]. Neither the assessment of correlations nor the logistic regression analysis could identify socioeconomic or psychosocial factors to have important influence on the GPs' assessment. This may indicate that these factors are beyond the scope of physicians, even if the GP, who is more familiar with the patient and his individual situation than all other physicians, is estimating patients' QoL. This is in accordance with findings of previous studies which, for instance, revealed that psychological factors as well as concomitant depressions are often missed by physicians treating OA [10]. In ignoring these factors, GPs could also miss the possibility to involve additional important caregivers or persons out of patients' social context such as a spouse or other family members and friends [33].

Our study suggests that instead of considering these important factors, GPs assessment of QoL is more focused on evident structural changes as documented in radiographs. Though it has been known for a long time that radiological findings show only poor correlation to pain and patients QoL, physicians' estimations are still strongly influenced by radiographs. Ignoring psychosocial influence factors may cause a lack of treatment and on the other hand considering factors which are less related to patients' QoL – as radiological findings – may lead to inadequate treatment. The implications for practice are obvious: our results suggest that physicians should consider physiological and social factors more intensely when treating patients suffering from OA. They should be aware that these factors contribute substantially to patients' QoL and may represent an important target for non-surgical and non-pharmacological interventions. Moreover, GPs should avoid overestimation of x-ray findings and treat patients not pictures.

## Competing interests

The author(s) declare that they have no competing interests.

## Authors' contributions

TR conceived and performed the study and drafted the manuscript. GL performed the statistical calculations. SJ and TK contributed substantially to the manuscript. JS participated in the study design. All authors read and approved the final manuscript.

## Pre-publication history

The pre-publication history for this paper can be accessed here:



## Figures and Tables

**Table 1:** Baseline-characteristics of patients separated by sex

Sex	Mainly affected joint		N	Kellgren (Mean)	S.D.	Duration Mean	S.D.	Age	S.D.
	Hip	Knee							

w	37	60	97	2.85*	0.96	11.34**	6.29	43.76**	23.58
m	78	45	123	2.55*	0.74	8.78**	5.11	49.86**	13.30
Total	115	105	220	2.68	0.85	9.90	5.79	47.17	18.75

Level of significance (t-test for group comparison): *p < 0.05; **p < 0.01)

**Table 2:** Kellgren Score distribution according to joint

		Kellgren-Score				Total
			
		1	2	3	4	
Joint*	Hip	8	31	56	20	115
	Knee	11	38	39	17	105
Total		19	69	95	37	220

*Adjusted ANOVAs (gender, age) revealed no significant differences

**Table 3:** Descriptive statistics and score distributions for the different assessments according to affected joint

	Affected joint	Range	N	Mean	SD
Age	Hip	38–89	115	56.53	12.39
	Knee	25–83	105	58.54	12.02
BMI*	Hip	20.3–39.7	115	26.35	3.49
	Knee	19.6–41.1	105	27.95	4.82
Duration of OA**	Hip	1–23	115	9.39	4.56
	Knee	1–24	105	11.27	5.91

Level of significance: *p < 0.05; **p < 0.01

**Table 4:** Descriptive statistics and score distributions for the different assessments

Assessment	range		joint	N	Mean	SD	p
GP score	0–10		Hip	115	3.97	2.36	0.43
			Knee	105	4.23	2.48	
Kellgren-Score	1–4		Hip	115	2.77	0.82	0.13
			Knee	105	2.59	0.89	
AIMS2-SF	0–10	physical	Hip	114	3.61	1.57	0.15
			Knee	101	3.92	1.64	
		affect	Hip	115	4.20	1.32	0.40
			Knee	104	4.34	1.25	
		symptom	Hip	115	3.05	2.70	0.03
			Knee	105	3.88	2.95	
		social	Hip	114	5.04	2.00	0.58
			Knee	104	5.18	1.87	
		role	Hip	63	8.33	2.69	0.12
			Knee	46	7.41	3.41	
WOMAC	0–10	Pain	Hip	114	5.21	1.99	0.01
			Knee	103	5.93	2.07	
		Stiffness	Hip	115	4.96	1.97	0.08
			Knee	105	5.48	2.43	
		Function	Hip	115	5.59	2.30	0.23
			Knee	105	5.98	2.50	
		Overall	Hip	115	5.25	2.08	0.10
			Knee	105	5.75	2.36	

**Table 5:** Correlations (Spearman rho) of GP score and x-ray findings with AIMS2-SF and WOMAC

	AIMS2-SF					WOMAC		
	
	Physical	Affect	Symptom	Social	Role (n = 109)	Function	Stiffness	Pain
GP-Score	0.495**	0.370**	0.598**	0.161*	-0.165	0.358**	0.373**	0.557**
Kellgren-Score	0.328**	0.320**	0.419**	0.107	-0.214	0.227	0.302	0.303

Level of significance: *p < 0.05; **p < 0.01

**Table 6:** Mean scores for demographic subgroups

		Sex			Age			Education		
		Male	Female	P	< 60	>= 60	P	High	Low	P

N		123	97		83	137		125	95	
WOMAC	Pain	4.92	6.41	**	4.65	7.11	**	4.62	6.31	**
	Function	6.44	5.26	**	5.13	6.84	**	4.91	6.43	**
	Stiffness	5.59	6.01	**	4.65	6.12	**	4.36	5.86	**
	Global	4.90	6.23	**	8.86	6.52	**	4.65	6.12	**
GPs assessment		3.43	4.94	**	3.36	5.30	**	3.06	4.88	**
x-ray		2.55	2.85		2.58	2.84	**	2.45	2.86	**
AIMS	Physical	3.25	4.43	**	3.20	4.74	**	3.09	4.26	**
	Affect	3.83	4.81	**	3.95	4.79	**	3.90	4.55	**
	Symptom	2.32	4.57	**	2.16	5.22	**	1.83	4.44	**
	Social	4.08	5.02	**	4.05	5.23	**	4.16	8.28	*
	Role	1.74 (n = 74)	2.72 (n = 35)		2.06 (n = 97)	1.98 (n = 12)	**	5.25 (n = 63)	2.31 (n = 46)	

Level of significance: *p < 0.05; **p < 0.01

**Table 7:** Results of logistic regression analysis

	Variable	β^ MathType@MTEF@5@5@+=feaafiart1ev1aaatCvAUfKttLearuWrP9MDH5MBPbIqV92AaeXatLxBI9gBaebbnrfifHhDYfgasaacH8akY=wiFfYdH8Gipec8Eeeu0xXdbba9frFj0=OqFfea0dXdd9vqai=hGuQ8kuc9pgc9s8qqaq=dirpe0xb9q8qiLsFr0=vr0=vr0dc8meaabaqaciaacaGaaeqabaqabeGadaaakeaaiiGacuWFYoGygaqcaaaa@2E64@	P	95% CI
Socio-demographic factors	age	0.0555	0.6611	[-0.19, 0.30]
	sex	-0.0720	0.3530	[-0.22, 0.08]
	education	-0.0693	0.6552	[-0.37, 0.24]
AIMS2-SF	physical	-0.0272	0.8343	[-0.28, 0.23]
	affect	0.0521	0.7276	[-0.24, 0.34]
	symptoms	0.2588	0.0267	[0.03, 0.49]
	social	-0.0107	0.9060	[-0.19, 0.17]
WOMAC	function	0.1235	0.2928	[-0.11, 0.35]
	stiffness	-0.0526	0.6726	[-0.30, 0.19]
	pain	0.1389	0.3545	[-0.16, 0.43]
x-ray	Kellgren-Score	0.6395	0.0004	[0.29, 0.99]
